# Author Correction: Whole genome sequencing of metastatic colorectal cancer reveals prior treatment effects and specific metastasis features

**DOI:** 10.1038/s41467-021-23629-4

**Published:** 2021-05-26

**Authors:** Pauline A. J. Mendelaar, Marcel Smid, Job van Riet, Lindsay Angus, Mariette Labots, Neeltje Steeghs, Mathijs P. Hendriks, Geert A. Cirkel, Johan M. van Rooijen, Albert J. Ten Tije, Martijn P. Lolkema, Edwin Cuppen, Stefan Sleijfer, John W. M. Martens, Saskia M. Wilting

**Affiliations:** 1 Department of Medical Oncology, Erasmus MC Cancer Institute, Erasmus University Medical Center Rotterdam, Rotterdam, The Netherlands; 2 Cancer Computational Biology Center, Erasmus MC Cancer Institute, Erasmus University Medical Center Rotterdam, Rotterdam, The Netherlands; 3 Department of Urology, Erasmus MC Cancer Institute, Erasmus University Medical Center Rotterdam, Rotterdam, The Netherlands; 4 Department of Medical Oncology, Cancer Center Amsterdam, Amsterdam UMC, Vrije Universiteit Amsterdam, Amsterdam, The Netherlands; 5 Center for Personalized Cancer Treatment, Rotterdam, The Netherlands; 6 Department of Medical Oncology, The Netherlands Cancer Institute, Antoni van Leeuwenhoek, Amsterdam, The Netherlands; 7 Department of Medical Oncology, Northwest Clinics, Alkmaar, The Netherlands; 8 Department of Medical Oncology, Meander Medical Center, Amersfoort, The Netherlands; 9 Department of Medical Oncology, Martini Hospital, Groningen, The Netherlands; 10 Department of Medical Oncology, Amphia Hospital, Breda, The Netherlands; 11 Center for Molecular Medicine and Oncode Institute, University Medical Center Utrecht, Utrecht, The Netherlands; 12 Hartwig Medical Foundation, Amsterdam, The Netherlands

**Keywords:** Cancer genomics, Colorectal cancer, Metastasis

Correction to: *Nature Communications* 10.1038/s41467-020-20887-6, published online 25 January 2021.

The original version of this Article contained an error in Table 4, in which the coefficients of the LASSO regression model of treatment response corresponded to a version that was performed without non-coding genes. The new version of the table, which was generated during revision of the manuscript, contains the coefficients that were obtained after including potential non-coding driver genes in the model. Genomic features that became statistically significant after re-running the model were also added, which included: ‘nr of 10 kb–1 Mb deletions’, ‘SBS41’, ‘Non-coding - LINC00672’, ‘Gain 7p12.3 - (PKD1L1)’, ‘Loss 4q22.1 - (CCSER1)’, and ‘Loss 18q23 - (NFATC1*)’. This has now been corrected in both the PDF and HTML versions of the Article.

The original version of this Article also contained an error in the author affiliations. The affiliations of Job van Riet with Cancer Computational Biology Center, Erasmus MC Cancer Institute, Erasmus University Medical Center Rotterdam, Rotterdam, The Netherlands and Department of Urology, Erasmus MC Cancer Institute, Erasmus University Medical Center Rotterdam, Rotterdam, The Netherlands were inadvertently omitted. This has now been corrected in both the PDF and HTML versions of the Article.

The original version of this Article contained an error in the Methods, section “Whole-genome sequencing; identification of somatic changes”, which incorrectly read ‘GATK BQSR and Haplotype Caller v3.4.46 were used to call somatic mutations.’ The correct version is ‘GATK BQSR and Haplotype Caller v3.4.46 and Strelka v1.0.14 were used to call somatic mutations’. This has been corrected in both the PDF and HTML versions of the Article.

