# Supplementary material for: Whole genome sequencing of metastatic colorectal cancer reveals prior treatment effects and specific metastasis features
Source: Nat Commun. 2021 Jan 25;12:574. doi: 10.1038/s41467-020-20887-6 (PMC7835235; doi:10.1038/s41467-020-20887-6)
Supplement: Supplementary file 1 — Supplementary Information [file 41467_2020_20887_MOESM1_ESM.pdf]

Whole genome sequencing of metastatic colorectal cancer reveals prior treatment effects and specific metastasis features

Supplementary Figures & Tables

Supplementary Figure 1: Mutual exclusivity in driver genes

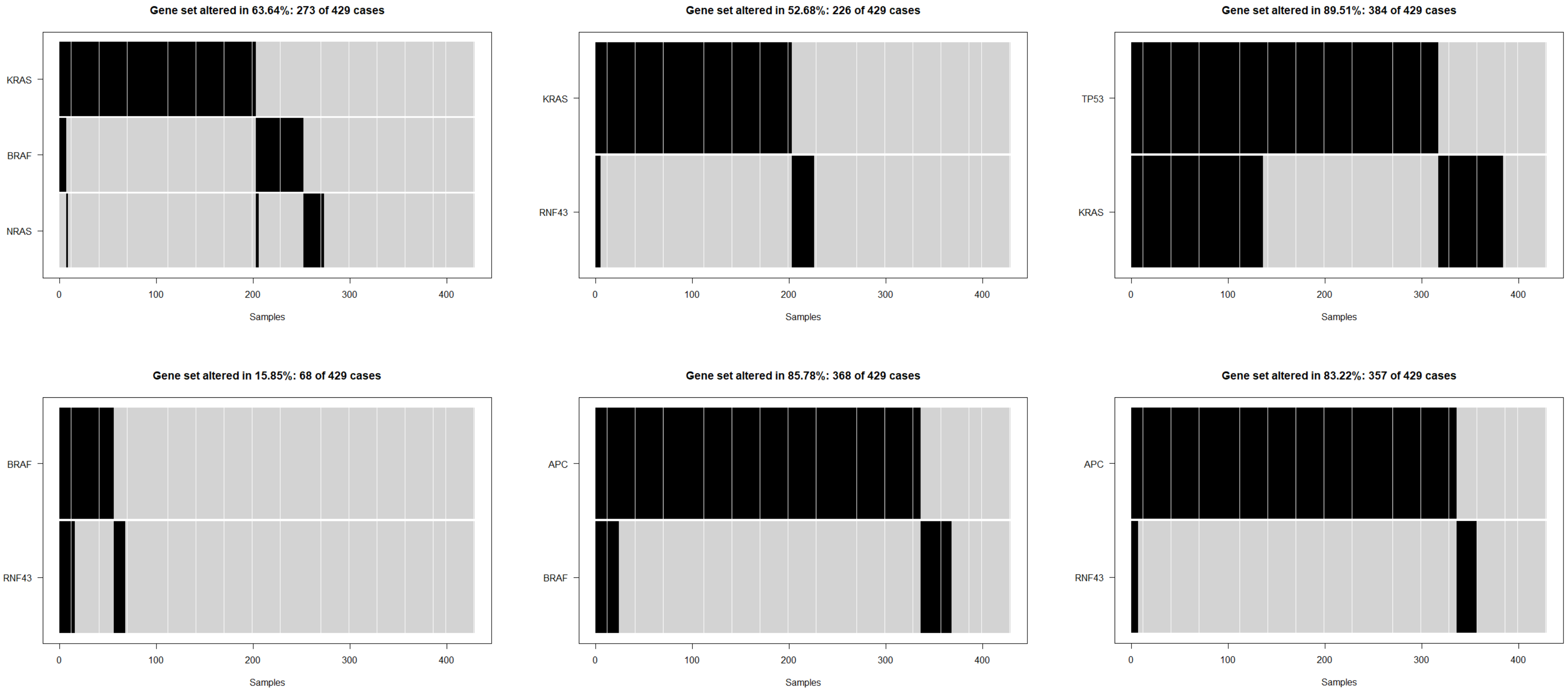

Supplementary Figure 1: Mutual exclusivity in driver genes

For the indicated genes, the number of mutated metastatic CRC cases are depicted in black. The R package discover v0.9.243 was used to test for mutual exclusivity: KRAS with BRAF/NRAS/RNF43/TP53 (q=1.06E-7, q=1.54E-4, q=0.004 and q=0.017, respectively), and APC with RNF43/BRAF (both q=1.54E-4).

Supplementary Figure 2: TIL score over MSI/TMB

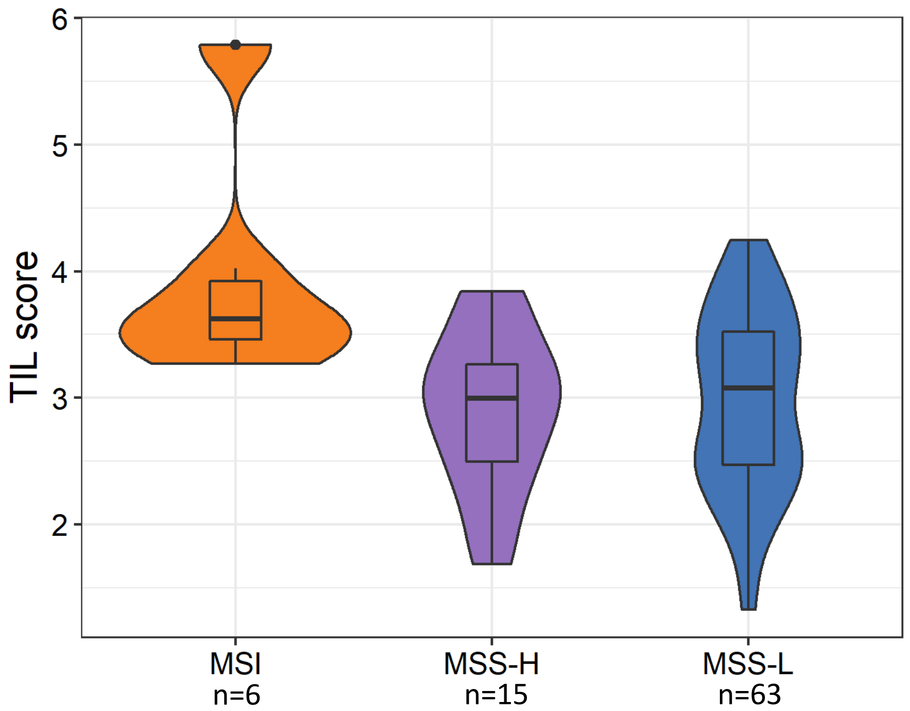

Supplementary Figure 2: TIL score over MSI/TMB

Tumor infiltrating lymphocytes (TIL) score by Microsatellite Instable (MSI, n=6 or Stable (MSS, n=78) groups, the latter divided by tumor mutational burden (TMB) status: MSS-H (n=15) indicates a TMB>10, MSS-L (n=63) <10 per Mb. The box shows the 1st to 3rd quartile, the horizontal bar shows the median and whiskers extend to 95% of the distribution. The Kruskal-Wallis test was significant (p=0.037) with the subsequent post-hoc analysis by Dunn's pairwise comparison resulting in Benjamini-Hochberg corrected p=0.012 and p=0.021 for MSI compared to MSS with high (MSS-H) and low TMB (MSS-L), respectively.

Supplementary Table 1: Public cohorts

Name	Mode	Type	N	Purpose
TCGA-DFCI	DNA	primary CRC	1949	compare gene mutation frequencies
PCAWG	WGS	primary CRC	73	compare mutational signatures
Yaeger	DNA-targeted	metastatic CRC	321	compare gene mutation frequencies
ICGC	DNA	primary CRC	866	compare non-coding mutation frequencies

Supplementary Table 2: Chromosomal regions vs. prior treatment

Prior treatment details										
Type of systemic prior treatment		Untreated n=124	Any pretreatment n=284	PLAT/PYR n=121	PLAT/PYR+targeted n=134	PYR n=39	PYR+targeted n=36	targeted n=35	TOP n=67	TOP/PYR n=26
**Chromosomal details**										
Alteration	*Cytoband*	*Frequency of alteration (%)*								
Amplification Peak 3	6p22.1	34.7	55.6	58.7	60.4	51.3	50.0	51.4	55.2	61.5
Amplification Peak 4	6p21.1	36.3	58.8	60.3	62.7	61.5	47.2	62.9	52.2	73.1
Amplification Peak 13	11p15.5	31.5	44.4	42.1	47.8	35.9	52.8	48.6	58.2	42.3
Amplification Peak 22	18p11.32	11.3	30.6	34.7	31.3	33.3	30.6	20.0	32.8	30.8
Deletion Peak 3	3p14.2	48.4	63.7	62.8	64.2	59.0	50.0	60.0	67.2	61.5
Deletion Peak 8	5q12.1	40.3	53.5	52.9	57.5	51.3	58.3	77.1	58.2	61.5
Deletion Peak 11	8p21.3	69.4	82.4	78.5	90.3	74.4	80.6	82.9	86.6	84.6
Deletion Peak 24	20p12.1	46.0	58.1	57.9	64.9	69.2	55.6	71.4	56.7	53.8

* Significantly differential alteration frequencies compared to untreated patients are depicted in red (Chi-square test; FDR<0.05)

Supplementary Table 3: Genes with decreased mutation

Gene	Primary TCGA		Meta		Fisher	FDR	% Change
	N	%	N	%	p-value	Hochberg	in meta
FCGBP	193	9.9	1	0.2	2.64E-16	1.22E-13	-9.7
MACF1	231	11.9	9	2.1	5.22E-12	2.42E-09	-9.8
GPR179	111	5.7	1	0.2	4.35E-09	2.00E-06	-5.5
MUC17	239	12.3	20	4.7	8.64E-07	3.97E-04	-7.6
AHNAK2	247	12.7	22	5.1	1.96E-06	9.01E-04	-7.5
FLG	363	18.6	41	9.6	2.42E-06	0.001	-9.1
DIDO1	168	8.6	11	2.6	2.52E-06	0.001	-6.1
IGF2R	132	6.8	7	1.6	6.37E-06	0.003	-5.1
AXIN2	132	6.8	7	1.6	6.37E-06	0.003	-5.1
FAM47C	122	6.3	6	1.4	8.36E-06	0.004	-4.9
FLNB	137	7.0	8	1.9	0.00001001	0.005	-5.2
BRWD3	108	5.5	5	1.2	0.00002402	0.011	-4.4
COL5A1	175	9.0	14	3.3	0.00002789	0.013	-5.7
RP1L1	151	7.7	11	2.6	0.00003062	0.014	-5.2
NFASC	133	6.8	9	2.1	0.00006445	0.029	-4.7
ANK3	194	10.0	18	4.2	0.00007292	0.033	-5.8
CHD4	160	8.2	13	3.0	0.00007946	0.036	-5.2
PCDHA9	107	5.5	6	1.4	0.00008121	0.036	-4.1
PDE4DIP	151	7.7	12	2.8	0.00008232	0.037	-5.0
TENM1	199	10.2	19	4.4	0.00009019	0.040	-5.8
TEX15	138	7.1	10	2.3	0.00009045	0.040	-4.7

P-values are derived from the Fisher's exact test (2-sided) and corrected for multiple testing using the FDR (Hochberg) method.

Supplementary Table 4: MSI Genes

Gene	Fisher p-value	Permutation p-value (1000 permutations)	FDR Hochberg	MSI Mutations (N) All	MSI Mutations (N) Frameshift	Total Mutations (N) All	Total Mutations (N) Frameshift	Known target gene of MSI (Kodelin et al, Cancer Res 2017; 77(15); 4078-88) chr	position (hg19)	context	seqcontext
ACVR2A	5.01E-14	0.001	1.61E-11	11	11	23	15	2	148683686	A8	catAAAAAAAAgag
UBR5	9.45E-13	0.002	3.02E-10	10	9	20	9	8	103289349	T8	ttcTTTTTTTTgcc
HERC2	1.46E-11	0.003	4.67E-09	11	3	35	4				
KMT2D	2.61E-11	0.001	8.30E-09	10	7	26	8				
KCNMA1	8.76E-11	0.002	2.78E-08	9	5	20	5				
RYR1	5.78E-10	0.01	1.83E-07	11	0	47	0				
UBR4	1.02E-09	0.009	3.21E-07	9	0	25	0				
TNRC18	2.30E-09	0.009	7.21E-07	9	0	27	0				
RNF213	3.35E-09	0.014	1.05E-06	9	3	28	5				
FSIP2	4.73E-09	0.041	1.48E-06	11	6	56	9				
NCAM1	5.35E-09	0.006	1.66E-06	8	7	20	7				
STAB2	5.35E-09	0.005	1.66E-06	8	0	20	0				
KMT2C	6.82E-09	0.017	2.11E-06	9	5	30	8				
LRP2	1.08E-08	0.042	3.32E-06	10	5	44	7				
SPTBN4	1.33E-08	0.014	4.07E-06	8	0	22	0				
LAMA1	1.33E-08	0.009	4.07E-06	8	0	22	0				
ZNF469	6.21E-08	0.045	1.88E-05	8	5	26	5				
DOCK3	8.73E-08	0.049	2.63E-05	8	7	27	7				
FAT1	8.73E-08	0.04	2.63E-05	8	3	27	6				
TNXB	1.21E-07	0.044	3.55E-05	8	0	28	0				
KIF26B	1.21E-07	0.044	3.55E-05	8	0	28	0				
DNAH17	1.21E-07	0.049	3.55E-05	8	0	28	0				
NIPBL	2.23E-07	0.025	6.52E-05	7	0	20	0				
BTBD11	3.31E-07	0.048	9.53E-05	7	5	21	5				
LTBP2	3.31E-07	0.042	9.53E-05	7	5	21	5				
LRP1	3.31E-07	0.034	9.53E-05	7	0	21	0				
ADAMTS18	4.79E-07	0.042	1.37E-04	7	3	22	5				
AHNAK2	4.79E-07	0.047	1.37E-04	7	0	22	0				

P-values are derived from the Fisher's exact test, the permutation test, and corrected for multiple testing using the FDR (Hochberg) method.
