# Supplementary material for: Whole genome sequencing of metastatic colorectal cancer reveals prior treatment effects and specific metastasis features
Source: Nat Commun. 2021 Jan 25;12:574. doi: 10.1038/s41467-020-20887-6 (PMC7835235; doi:10.1038/s41467-020-20887-6)
Supplement: Supplementary file 3 — Description of Additional Supplementary Files [file 41467_2020_20887_MOESM3_ESM.pdf]

Description of Additional Supplementary Files

File Name: Supplementary Data 1
Description: List of Affected chromosomal regions (GISTIC)

File Name: Supplementary Data 2
Description: Prior treatment signatures

File Name: Supplementary Data 3
Description: Input data treatment outcome analysis
