# Supplementary material for: Whole genome sequencing of metastatic colorectal cancer reveals prior treatment effects and specific metastasis features
Source: Nat Commun. 2021 Jan 25;12:574. doi: 10.1038/s41467-020-20887-6 (PMC7835235; doi:10.1038/s41467-020-20887-6)
Supplement: Supplementary file 7 — Reporting Summary [file 41467_2020_20887_MOESM7_ESM.pdf]

nature research

Corresponding author(s): S. Wilting / M.Smid / P.A.J. Mendelaar /J.W.M. Martens

Last updated by author(s): 19-12-2020

Reporting Summary

Nature Research wishes to improve the reproducibility of the work that we publish. This form provides structure for consistency and transparency in reporting. For further information on Nature Research policies, see our Editorial Policies and the Editorial Policy Checklist.

Statistics

For all statistical analyses, confirm that the following items are present in the figure legend, table legend, main text, or Methods section.

n/a	Confirmed	
☐	☒	The exact sample size (*n*) for each experimental group/condition, given as a discrete number and unit of measurement
☒	☐	A statement on whether measurements were taken from distinct samples or whether the same sample was measured repeatedly
☐	☒	The statistical test(s) used AND whether they are one- or two-sided<br>*Only common tests should be described solely by name; describe more complex techniques in the Methods section.*
☐	☒	A description of all covariates tested
☐	☒	A description of any assumptions or corrections, such as tests of normality and adjustment for multiple comparisons
☐	☒	A full description of the statistical parameters including central tendency (e.g. means) or other basic estimates (e.g. regression coefficient) AND variation (e.g. standard deviation) or associated estimates of uncertainty (e.g. confidence intervals)
☐	☒	For null hypothesis testing, the test statistic (e.g. *F*, *t*, *r*) with confidence intervals, effect sizes, degrees of freedom and *P* value noted<br>*Give P values as exact values whenever suitable.*
☒	☐	For Bayesian analysis, information on the choice of priors and Markov chain Monte Carlo settings
☒	☐	For hierarchical and complex designs, identification of the appropriate level for tests and full reporting of outcomes
☐	☒	Estimates of effect sizes (e.g. Cohen's *d*, Pearson's *r*), indicating how they were calculated

Our web collection on statistics for biologists contains articles on many of the points above.

Software and code

Policy information about availability of computer code

Data collection	DNA:HiSeqX (V2.5 reagents), bcl2fastq tool (versions 2.17 to 2.20) Illumina. BWA-mem v0.7.5a. GATK BQSR and Haplotype Caller v3.4.46<br>RNA: NextSeq 500 (V2.5 reagents), bcl2fastq (v2.17-2.20) fastp (v0.20.0) STAR (v2.6.1d), Sambamba (v0.7.0) featureCounts (v1.6.3)
Data analysis	All tools and settings are described here: https://github.com/hartwigmedical. Open sources: Strelka v1.0.14. Manta v1.0.3. R version 3.6.0. GISTIC2.0 v2.0.23, dndscv v0.0.0.9, tilingarray v1.56.0, karyoploteR v.1.4.1, MutationalPatterns v1.10.0, Discover v0.9.2, NMF v0.21.0, seriation v1.2.3 Shatterseek v0.4 CMSclassifier v1.0.0, CMSCaller v0.99.1, MASS v7.3-52.4, ordinalNet v2.7. Commercial SPSS v24, STATA13.0

For manuscripts utilizing custom algorithms or software that are central to the research but not yet described in published literature, software must be made available to editors and reviewers. We strongly encourage code deposition in a community repository (e.g. GitHub). See the Nature Research guidelines for submitting code & software for further information.

Data

Policy information about availability of data

All manuscripts must include a data availability statement. This statement should provide the following information, where applicable:

- Accession codes, unique identifiers, or web links for publicly available datasets
- A list of figures that have associated raw data
- A description of any restrictions on data availability

Data is available at the Hartwig Medical Foundation and can be requested upon through the following link: https://www.hartwigmedicalfoundation.nl/applying-for-data/ (request number: DR-058)Publicly available datasets that were used in this study are listed in Supplementary Table 1.

Field-specific reporting

Please select the one below that is the best fit for your research. If you are not sure, read the appropriate sections before making your selection.

- [x] Life sciences
- [ ] Behavioural & social sciences
- [ ] Ecological, evolutionary & environmental sciences

For a reference copy of the document with all sections, see nature.com/documents/nr-reporting-summary-flat.pdf

Life sciences study design

All studies must disclose on these points even when the disclosure is negative.

Sample size	No sample size was calculated, all data of the available cohort was requested and analyzed
Data exclusions	Patients whose treatment data did not match standard of care for colorectal cancer were excluded to avoid erroneous inclusion of patients suffering from another type of cancer (n = 28). When multiple biopsies were included for one patient (n = 29), only the first biopsy was included in our analyses.
Replication	No replication experiments were possible in our study; a single biopsy per affected organ was taken for analysis per patient.
Randomization	Randomization was not relevant to our study, because this is a descriptive, prospective cohort study, not a clinical trial
Blinding	Blinding was not relevant to our study, because this is a descriptive, prospective cohort study, not a clinical trial

Reporting for specific materials, systems and methods

We require information from authors about some types of materials, experimental systems and methods used in many studies. Here, indicate whether each material, system or method listed is relevant to your study. If you are not sure if a list item applies to your research, read the appropriate section before selecting a response.

Materials & experimental systems

n/a	Involved in the study	
x		Antibodies
x		Eukaryotic cell lines
x		Palaeontology and archaeology
x		Animals and other organisms
	x	Human research participants
	x	Clinical data
x		Dual use research of concern

Methods

n/a	Involved in the study	
x		ChIP-seq
x		Flow cytometry
x		MRI-based neuroimaging

Human research participants

Policy information about studies involving human research participants

Population characteristics	Patients with metastatic solid cancer above the age of 18, starting a new line of systemic treatment (irrespective of disease stage or number of treatment lines). Clinical characteristics are available in Table 1.
Recruitment	Patients were included via the outpatient medical oncology clinics of different hospitals in The Netherlands
Ethics oversight	Medical ethical board of the University Medical Center Utrecht (UMCU), local approval by the direction board of the Erasmus Medical Center (EMC), Rotterdam

Note that full information on the approval of the study protocol must also be provided in the manuscript.

Clinical data

Policy information about clinical studies

All manuscripts should comply with the ICMJEguidelines for publication of clinical research and a completedCONSORT checklist must be included with all submissions.

Clinical trial registration	NCT01855477
Study protocol	https://clinicaltrials.gov/ct2/show/study/NCT01855477?term=CPCT&cntry=NL&draw=2&rank=1
Data collection	Upon our data request, Hartwig Medical Foundation provided us with the data of all registered metastatic CRC patients included between April 2016 and January 2019 (N=487).

Outcomes	As this is a descriptive cohort study, we did not have any defined endpoints.
